# The Unusual Metalloprotease-Rich Venom Proteome of the Australian Elapid Snake *Hoplocephalus stephensii*

**DOI:** 10.3390/toxins14050314

**Published:** 2022-04-28

**Authors:** Theo Tasoulis, C. Ruth Wang, Joanna Sumner, Nathan Dunstan, Tara L. Pukala, Geoffrey K. Isbister

**Affiliations:** 1 Clinical Toxicology Research Group Newcastle, University of Newcastle, Newcastle, NSW 2308, Australia; geoff.isbister@gmail.com; 2 Department of Chemistry, Faculty of Sciences, University of Adelaide, Adelaide, SA 5005, Australia; chia-de.wang@adelaide.edu.au (C.R.W.); tara.pukala@adelaide.edu.au (T.L.P.); 3 Genetic Resources, Museums Victoria, Carlton Gardens, Melbourne, VIC 5053, Australia; jsumner@museum.vic.gov.au; 4 Venom Supplies, Tanunda, SA 5352, Australia; venoms@venomsupplies.com

**Keywords:** elapid, Australian elapids, *Hoplocephalus*, snake venom, snake venom proteome, snake venomics

## Abstract

The Australasian region is home to the most diverse elapid snake radiation on the planet (Hydrophiinae). Many of these snakes have evolved into unique ecomorphs compared to elapids on other continents; however, their venom compositions are poorly known. The Australian elapid *Hoplocephalus stephensii* (Stephen’s banded snake) is an arboreal snake with a unique morphology. Human envenoming results in venom-induced consumption coagulopathy, without neurotoxicity. Using transcriptomics and a multi-step fractionation method involving reverse-phase high-performance liquid chromatography, sodium dodecyl sulfate polyacrylamide gel electrophoresis and bottom-up proteomics, we characterized the venom proteome of *H. stephensii.* 92% of the total protein component of the venom by weight was characterized, and included all dominant protein families and 4 secondary protein families. Eighteen toxins made up 76% of the venom, four previously characterized and 14 new toxins. The four dominant protein families made up 77% of the venom, including snake venom metalloprotease (SVMP; 36.7%; three identified toxins), phospholipase A_2_ (PLA_2_; 24.0%; five identified toxins), three-finger toxin (3FTx; 10.2%; two toxins) and snake venom serine protease (SVSP; 5.9%; one toxin; Hopsarin). Secondary protein families included L-amino acid oxidase (LAAO; 10.8%; one toxin), natriuretic peptide (NP; 0.8%; two toxins), cysteine-rich secretory protein (CRiSP; 1.7%; two toxins), c-type lectin (CTL; 1.1%; one toxin), and one minor protein family, nerve growth factor (NGF; 0.8%; one toxin). The venom composition of *H. stephensii* differs to other elapids, with a large proportion of SVMP and LAAO, and a relatively small amount of 3FTx. *H. stephensii* venom appeared to have less toxin diversity than other elapids, with only 18 toxins making up three-quarters of the venom.

## 1. Introduction

**Figure 1:**
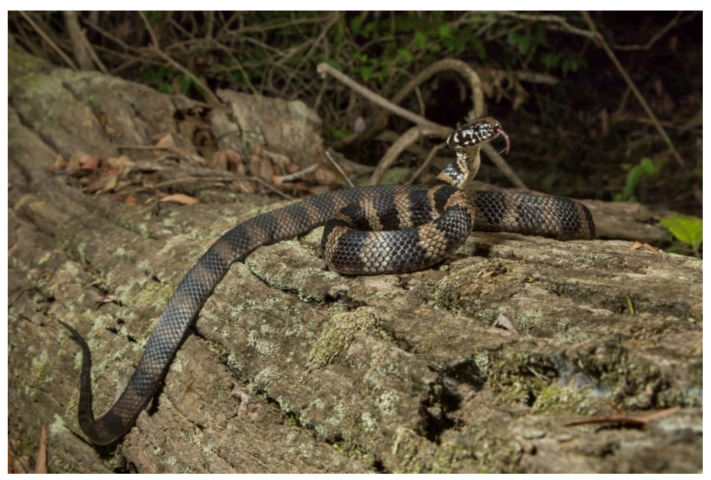
*Hoplocephalus stephensii*, showing the thin neck, large flat head and body proportions which are a unique morphology for an elapid. Photo courtesy of Brendan Schembri.

Of the three families of front-fanged snakes occurring worldwide [1,2], only Elapidae occurs on the Australian continent. The Australasian region is home to the most diverse elapid snake radiation on the planet (Hydrophiinae). Many of these snakes have evolved into unique ecomorphs compared to elapids on other continents. Within Hydrophiinae, the genus *Hoplocephalus* belongs to the live-bearing *Notechis* clade, which contains the medically significant species tiger snake (*Notechis scutatus*) and rough-scaled snake (*Tropidechis carinatus*) [3]. *Hoplocephalus* contains three morphologically similar species: the pale-headed snake *H. bitorquatus*, the broad-headed snake *H. bungaroides*, and Stephen’s banded snake *H. stephensii* (Figure 1). They are medium-sized (up to 1 metre in length) snakes occurring in eastern Australia and are almost entirely nocturnal, spending most of their lives inactive in shelter sites, moving infrequently to a new site [4,5,6]. Along with their sister genus *Paroplocephalus*, they are the only arboreal Australian elapids, and the only genera to have evolved specialized morphology for arboreality. In *Hoplocephalus*, this consists of distinctly keeled and notched ventral scales as an adaptation for climbing [7,8]. An interesting feature of their arboreality is that their use of hollow branches for shelter sites is largely restricted to eucalypt trees [4,9,10,11]. Despite being largely arboreal, they may be found active on the ground at night.

*H. stephensii* has been recorded in rainforest and wet and dry sclerophyll forest [4,5]. However, from 19 field observations by one of the authors (T.T.), none were in rainforest despite extensive searches in that habitat. They showed a preference for wet and dry sclerophyll forest that were adjacent, and individuals were located from the top of the dry sclerophyll ridges to the bottom of the wet sclerophyll gullies. Juvenile *H. stephensii* feed on lizards while adults feed primarily on mammals [6]. TT has made two opportunistic observations of adult *H. stephensii* preying on adult agamid lizards in the wild. One individual was swallowing a Southern angle-headed dragon *Lophosaurus spinipes* on the ground during the day (mid-April), which may indicate a switch to diurnal hunting during the cooler autumn months or may merely have been an opportunistic interaction. The second individual was scent tracking a bitten and immobilized Jacky lizard *Amphibolururs muricatus* on the ground at night at 0400 h (December), the lizard was presumably bitten while sleeping on the branch of a small tree.

Previous studies on *H. stephensi* venom have shown that it possesses procoagulant [12] and in vitro post-synaptic neurotoxic activity [13]. The toxins responsible for these activities have been identified and purified. The procoagulant is a serine protease (SVSP), namely a Factor Xa homologue (FXa) called Hopsarin D [14,15], which is the catalytic subunit of the prothrombinase complex. It consists of two subunits with molecular masses of 23 and 36 kDa. The toxin responsible for in vitro post-synaptic neurotoxic activity is a short-chain three-finger toxin (3FTx), with a molecular mass of 6.66 kDa and has been named Hostoxin-1 [16]. The main clinical feature of bites by *H. stephensii* is venom-induced consumption coagulopathy (VICC), and rarely thrombotic microangiopathy, but not neurotoxicity or myotoxicity [17,18].

Although approximately 50 different protein families have been identified in snake venoms worldwide, most snake venoms have been shown to be largely composed of four dominant protein families; three-finger toxins (3FTx), phospholipase A_2_ (PLA_2_), snake venom metalloprotease (SVMP), snake venom serine protease (SVSP), and six secondary protein families; L-amino acid oxidase (LAAO), kunitz peptides (KUN), cysteine-rich secretory proteins (CRiSP), natriuretic peptides (NP), C-type lectins (CTL), and disintegrins (DIS) [19,20].

Despite the long history of research in characterizing the pharmacology of toxins in the venoms of the Hydrophiinae subfamily, the venom composition of the species in this subfamily remains the least characterized of any group of elapids or vipers [19,20]. We aimed to determine the venom composition of Stephen’s banded snake *H. stephensii* to investigate venom expression in a morphologically and ecologically specialized elapid.

## 2. Results

### 2.1. Transcriptomics

The toxin genes made up 1851 of the total 37,881 genes in the venom gland transcriptome (5%), and 39% of the gene expression (353,834 versus 549,862 normalized reads). Based on a keyword search of published snake toxin protein families, the functionally annotated genes in the venom gland were assigned to 28 recognized toxin families [19]: PLA_2_, 3FTx, SVMP, SVSP, CRiSP, LAAO, NP, DIS, KUN, CTL, endonuclease, aminopeptidase, aminotransferase, kinesin-like, inositol polyphosphate phosphatase, transferrin, kazal-type inhibitor, insulin-like growth factor, type-b carboxylesterase, endopeptidase, cholinesterase, peroxiredoxin, aspartic protease, sulfhydryl oxidase glutaminyl cyclase, selectin, neurotrophin and prokineticin.

**Figure 2:**
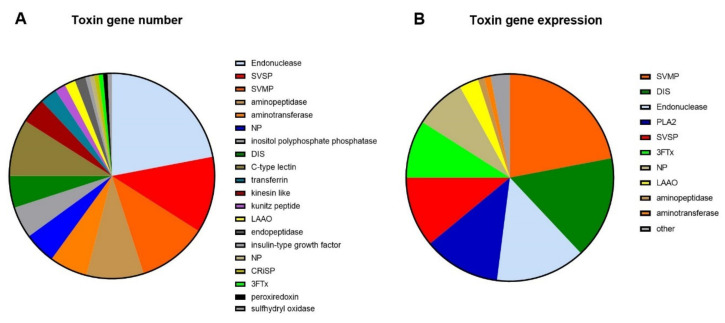
(**A**) Number of toxin genes in each toxin family from the venom gland transcriptome of *Hoplocephalus stephensii*. (**B)** Representation of the toxin gene expression in the venom gland transcriptome (counts per million—CPM).

Nine protein families made up 84% of toxin gene diversity in the *H. stephensii* venom gland transcriptome (Figure 2A), including endonuclease (22%), SVSP (12%), SVMP (11%), CTL (9%), aminopeptidase (9%), aminotransferase (6%) PLA_2_ (5%), DIS (5%) and inositol polyphosphate phosphatase (5%). The expression levels of the toxin protein families differed markedly, with just eight of the 25 protein families making up 95% of the transcriptome toxin gene expression (Figure 2B). This included all four primary toxin families—SVMP (22%), PLA_2_ (12%), SVSP (11%), and 3FTx (9%); and three secondary protein families—DIS (16%), NP (8%) and LAAO (3%). The only highly expressed protein family not belonging to either of these two groups was endonuclease (14%).

### 2.2. Proteomics

#### 2.2.1. Separation of the whole venom by Reverse-Phase High-Performance Liquid Chromatography (RP-HPLC)

**Figure 3:**
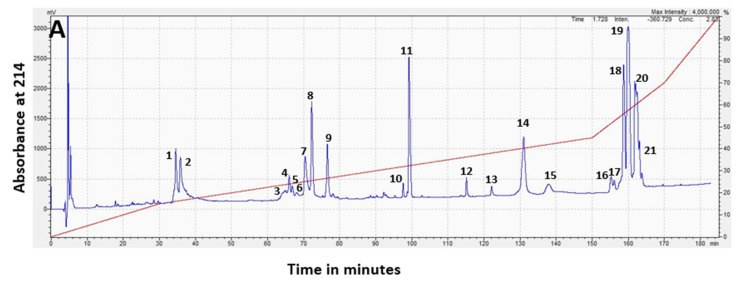

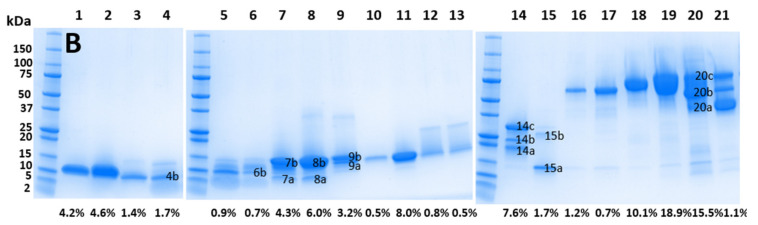
(**A**) RP-HPLC chromatogram of pooled *Hoplocephalus stephensii* venom with peaks numbered, and acetonitrile gradient superimposed in red. The *x* axis is time in minutes, and the *y* axis is the absorbance at 214 nm. The venom was reconstituted in water, and the early eluting high peak before 10 min is the non-protein-containing injection peak. (**B**) SDS-PAGE analysis of the venom fractionated by RP-HPLC with corresponding chromatogram peak numbers above panel, and percentages of the whole venom below panel. Numbers on the Y-axis show the apparent molecular mass of the protein standards in kDa. Multiple bands sampled in a single lane are labelled for cross-reference with entries in Table 1 and Table 2.

RP-HPLC separation of *H. stephensii* venom produced a chromatogram with 21 prominent peaks (each of these prominent peaks being >0.5% of the whole venom), eluting from 33 to 166 min (Figure 3A). Forty five percent of the venom (peaks 18 to 21) was hydrophobic, eluting after the acetonitrile gradient exceeded 50%. Visualization of the venom fractions corresponding to these chromatographic peaks by gel electrophoresis (Figure 3B) showed that peaks 1 to 13, which made up approximately 37% of the whole venom, were most intensely stained in the mass range of 10–15 kDa. Peak 14 showed three bands in the mass range of 20–35 kDa. Peaks 16 to 21, which made up 47% of the whole venom, was comprised primarily of higher-molecular-weight components with intense staining in the mass range of 40–100 kDa.

#### 2.2.2. Protein Family Identification and Quantification

We used PEAKS Xpro software (Bioinformatics Solutions Inc. Waterloo, ON, Canada), to match the peptide sequences derived from bottom-up proteomics to complete protein sequences on our assembled species-specific transcriptome. Using a BLAST search on UniProt, the protein sequences were then matched to either a protein family based on a high sequence similarity to a known toxin, or in some cases to a specific toxin if known (i.e., sequence available in UniProt). The identified protein families for each chromatographic peak are shown in Table 1. The relative abundance of each protein family was calculated by either integration of chromatogram peaks, or a combination of peak integration, densitometry and MSI spectral intensity. (Table 1 and  Supplementary file S1).

**Figure 4:**
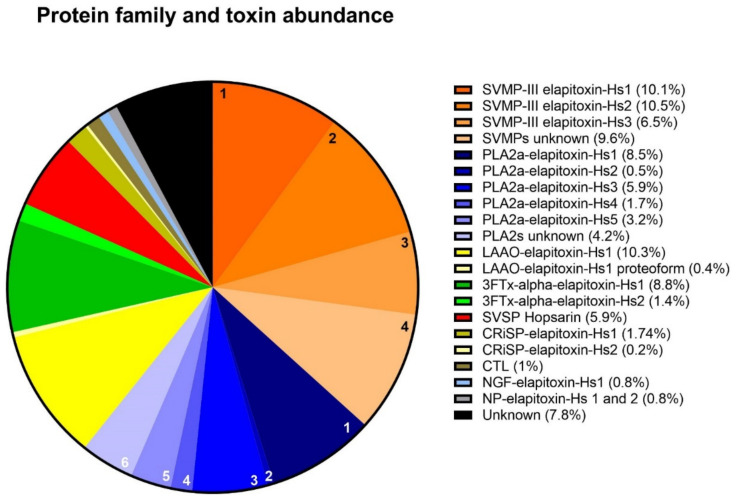
Venom proteome of *Hoplocephalus stephensii*. The legend shows the abundance of each toxin in the venom proteome of *H. stephensii* grouped into their respective protein families. Orange sectors: (1), SVMP-III elapitoxin-Hs1 (10.1%), (2), SVMP-III elapitoxin-Hs2 (10.5%), (3), SVMP-III elapitoxin-Hs3 (6.5%), and (4), unknown SVMPs (9.6%). Blue sectors: (1), PLA2a-elapitoxin-Hs1 (8.5%), (2), PLA2a-elapitoxin-Hs2 (0.5%), (3), PLA2a-elapitoxin-Hs3 (5.9%), (4), PLA2a-elapitoxin-Hs4 (1.7%), (5), PLA2a-elapitoxin-Hs5 (3.2%), and (6), unknown PLA_2_s (4.2%). Yellow sectors: LAAO-elapitoxin-Hs1 (10.3%, yellow), and LAAO-elapitoxin-Hs1 proteoform (0.4%, cream). Green sectors: 3FTx, alpha-elapitoxin-Hs1 (8.8%, dark green), and alpha-elapitoxin-Hs2 (1.4%, light green). Red sector: SVSP, Hopsarin (5.9%). Olive green sectors: CRiSP elapitoxin-Hs-1 (1.5% dark) and CRiSP-elapitoxin-Hs-2 (0.2%, light). Brown sector: CTL (1%). Light blue sector: NGF-elapitoxin-Hs-1 (0.8%). Grey sector: NP-elapitoxin-Hs-1 and NP-elapitoxin-Hs-2 (0.8%). Black sector: Unknown toxins (7.8%).

Proteomic analysis of the excised gel bands identified all four of the dominant protein families, four of the six secondary protein families (LAAO, CRiSP, CTL and DIS) and one minor protein family (nerve growth factor [NGF]). Integration of the identified chromatogram peaks showed that SVMP was the most abundant toxin family in the *H. stephensii* venom proteome, making up 36.7% of the venom (Figure 4). The SVMP were from class P-III containing a disintegrin domain, so we have quantified these two protein families together as SVMP in our venom proteome image (Figure 4). The second most abundant protein family was PLA_2_ (24.0%), and then in decreasing order, LAAO (10.8%), 3FTx (10.2%), SVSP (5.9%), CRiSP (1.7%), CTL (1.1%), and NGF (0.8%). A row of faintly stained low-molecular-weight bands in peaks 4 through to 6 were not analyzed due to their small percentage of the whole venom and their low protein content. Combined peak integration and densitometry calculations indicated that these low-molecular-weight proteins (<5 kDa) made up 0.8% of the whole venom. Whole-venom proteomic analysis identified NP in the venom, which are likely to be the low-molecular-weight bands in peaks 4 to 6 (Supplementary File 2, Table S1). Additionally, NP has been recorded staining gels at this mass region in the closely related tiger snake *N. scutatus* [21]. Whole-venom mass spectrometric analysis also identified sulfhydryl oxidase, 5′ nucleotidase, glutaminyl cyclase, hyaluronidase, phosphatase, phospholipase B, amininopeptidase and waprin (Supplementary file S2, Table S1).

#### 2.2.3. Toxin Identification and Amino Acid Sequences Deduced from the Transcriptome

PEAKS XPro software was used to match MS derived peptides with our transcriptome assembly. For each protein family identified above in a peak/band (Table 1), the derived amino acid sequence(s) were matched to identify the closest toxin in UniProt using a BLAST search. One or multiple derived amino acid sequences were then aligned and compared to previously identified toxins to determine the full amino acid sequence of each toxin in the toxin family.

**Figure 5:**
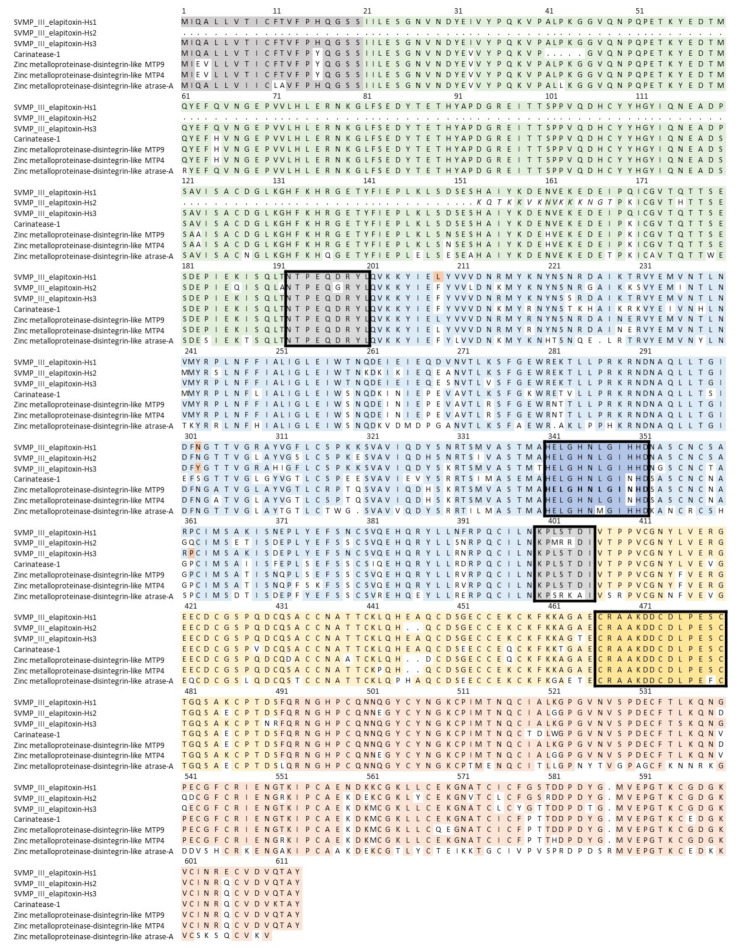
Deduced amino acid sequences for three new SVMP toxins found in *H. stephensii* venom, SVMP-III elapitoxin-Hs1, SVMP-III elapitoxin-Hs2 and SVMP-III elapitoxin-Hs3 (rows 1–3), compared to previously identified SVMPs from *T. carinatus* (B5KFV1; row 4), two from *Drysdalia coronoides* (F8RKV9; row 5; F8RKW1; row 6) and from *Naja atra* (D5LMJ3; row 7). The signal sequences are in shaded grey. The interdomain segments are shaded grey and boxed and the various domains of the precursor protein are: pro-domain, green; metalloprotease domain, blue; disintegrin domain, yellow; and cysteine-rich domain, red. The Zn^2+^ binding region in the metalloprotease domain is dark blue in a box and the conserved loop also found in ADAMs (dark yellow in a box).

From peaks 16 to 21, one complete and 18 partial amino acid sequences for SVMPs were deduced from MS/MS protein fragments matched to the transcriptome. These were aligned to the closest two matches in UniProt, zinc metalloproteinase-disintegrin-like MTP9 (F8RKV9) and zinc metalloproteinase-disintegrin-like MTP4 (F8RKW1), from *Drysdalia coronoides* using ALIGN. We were able to deduce sequences for three new SVMPs (Figure 5), which belonged to the P-III class. SVMP-III elapitoxin-Hs1 was in peak 18 and made up 10.1% of the venom, SVMP-III elapitoxin-Hs2 was in peak 19 and made up 10.5% of the venom and SVMP-III elapitoxin-Hs3 was in peak 20b and made up 6.5% of the venom (Table 1 and Table 2; Figure 5). The number of transcripts suggests that there are likely to be more toxins or proteoforms, but these could not be determined.

**Figure 6:**
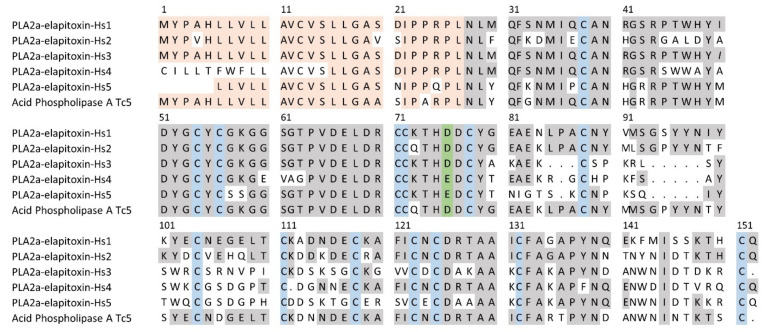
Deduced amino acid sequences for five new PLA_2_ toxins found in *H. stephensii* venom compared to the previously identified acid PLA_2_ in *T. carinatus* venom (acid phospholipase A Tc5; Q45Z26). The signal peptide is shaded light orange, conserved cysteine residues are shaded in blue, the active site in green and shaded grey areas indicate the same residue compared to PLA2a-elapitoxin-Hs1. PLA2a-elapitoxin-Hs4 and PLA2a-elapitoxin-Hs5 are uncommon variants of PLA_2_s, with a glutamate (E), replacing the Aspartate (D) in the active site.

There were 27 complete or partial amino acid sequences deduced from MS/MS protein fragments matched to the transcriptome for peaks containing PLA_2_ (Table 1). These were aligned using the closest matched acidic PLA_2_ in UniProt, acidic phospholipase A2 5 (Q45Z26) from *T. carinatus* venom. We deduced the amino acid sequence for five new acidic PLA_2_s, which ranged from 116 to 125 residues in length, with a signal peptide of 27 residues (Figure 6). No PLA_2_s have previously been identified in *H. stephensii* venom. PLA2a-elapitoxin-Hs1 was found in peaks 10 to 12 and made up 8.5% of the venom, PLA2a-elapitoxin-Hs2 in peak 9a, 0.5%, PLA2a-elapitoxin-Hs3, in peaks 8b and 9a, 5.9%, PLA2a-elapitoxin-Hs4 in peak 9b, 1.7% and PLA2a-elapitoxin-Hs5 in peak 7, 3.2% (Table 1 and Table 2; Figure 6). Peaks 4, 6, 7, 14a contained at least one other PLA2, making up 2.2% of the venom, but the sequences could not be determined.

There were two complete amino acid sequences deduced for LAAO from MS/MS protein fragments matched to the transcriptome, differing by only four residues. These were aligned using the closest matched LAAO in UniProt, L-amino-acid-oxidase (*N. scutatus*; Q4JHE2). Further examination of the peptide fragments matched to these deduced amino acid sequences suggests there is one LAAO in peak 19, LAAO-elapitoxin-Hs1, making up 8.3% of the venom. LAAO-elapitoxin-Hs1 also made up 2% of the venom in peak 20c, so the toxin made up 10.3% of the total venom (Figure 4). Peak 20a contains a small amount of LAAO (0.4%), which is most likely the same toxin or possibly a proteoform with four different residues (Table 1 and Table 2, Supplementary File S2, Figure S1).

**Figure 7:**
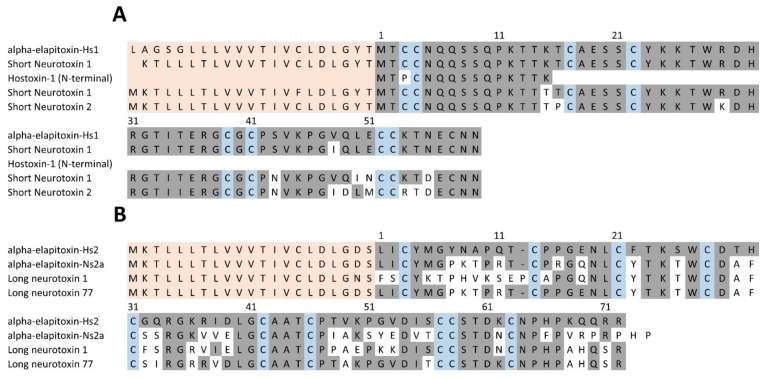
(**A**) Deduced amino acid sequence for the short-chain 3FTx (alpha-elapitoxin-Hs1) in *Hoplocephalus stephensii.* This deduced sequence (first row) is almost identical to two previously published sequences for this toxin from a BLAST search on UniPROT (short neurotoxin 1, A8HDJ9; row 2 and Hostoxin-1; row 3). Rows 4 and 5 are the previously published sequences for short-chain neurotoxins from the close relatives *Notechis scutatus* (short neurotoxin 1; A0A6J1VI58) and *Tropidechis carinatus* (short neurotoxin 2; A8HDJ6) from a BLAST search on UniPROT. (**B**) Deduced amino acid sequence for a new long-chain 3FTx (alpha-elapitoxin-Hs2; row 1) from the venom of *H. stephensii* compared to amino acid sequences of long neurotoxins from *N. scutatus* (alpha-elapitoxin-Ns2a; P01384; row 2), *T. carinatus* (long neurotoxin 1; A8HDK4; row 3) and *Drysdalia coronoides* (long neurotoxin 77; F8J2E2; row 4) from a BLAST search on UniPROT. The signal peptide is shaded light orange, conserved cysteine residues are shaded in blue and shaded areas indicate the same residue compared to alpha-elapitoxin-Hs2 and alpha-elapitoxin-Hs2 (B).

There were two complete amino acid sequences deduced for 3FTxs from MS/MS protein fragments matched to the transcriptome, one short-chain neurotoxin and long-chain neurotoxin. The short-chain neurotoxin made up 8.8% of the venom and was the only toxin in peaks 1 and 2; (Table 1 and Table 2). This short-chain neurotoxin (60 residues) has been previously identified and variously named Hostoxin-1 [16] and *H. stephensii* SNTX-1 (short neurotoxin) [22]. We have renamed this as alpha-elapitoxin-Hs1 (Figure 7). The long-chain neurotoxin has not been previously identified and has now been named alpha-elapitoxin-Hs2. It has 72 residues, the only toxin in peak 3 and made up 1.4% of the venom (Figure 7).

There were seven complete or partial amino acid sequences deduced for SVSP from MS/MS protein fragments matched to the transcriptome for peaks 14a and 14c (Table 1). A BLAST search on UniProt matched these all to the previously identified SVSP from *H. stephensii*, named Hopsarin D. Fragments from the proteins extracted from peak 14a only matched the light-chain part of the sequence, supporting the light chain of Hopsarin being in peak 14a (Supplementary File S2, Figure S2). Fragments from the proteins extracted from 14c only matched the heavy chain, supporting the heavy chain being in peak 14c (Supplementary File S2, Figure S2).

**Figure 8:**
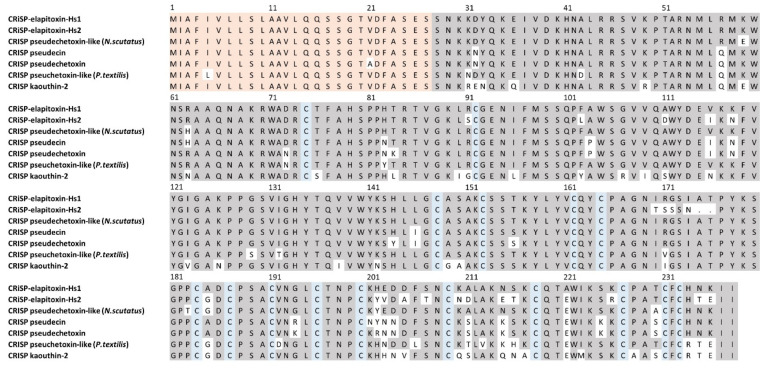
Deduced amino acid sequence for two cysteine-rich secretory proteins, the previously identified CRISP-elapitoxin-Hs1 (Q3SB03; row 1) and the new CRISP-elapitoxin-Hs2 (row 2) in the venom of *Hoplocephalus stephensii* compared to CRISPs found in the venoms of *Notechis scutatus* (Q3SB04; row 3), *Pseudechis porphyriacus* (Q8AVA3; row 4), *P. australis* (Q8AVA4; row 5), *Pseudonaja textilis* (Q3SB05; row 6) and *Naja kaouthia* (P84808; row 7). The signal peptide is shaded light orange (27 amino acids), conserved cysteine residues are shaded in blue and shaded areas indicate the same residue compared to CRISP-elapitoxin-Hs1.

There were two complete and six partial amino acid sequences deduced for CRiSP from MS/MS protein fragments matched to the transcriptome for peaks 14a, 14b and 15b (Table 1). A BLAST search on UniProt matched one of the complete sequences exactly to Cysteine-rich venom protein pseudechetoxin-like (*H. stephensii*). This was renamed as CRiSP-elapitoxin-Hs1 and was found in peaks 14a and 14b, making up 1.5% of the venom. A second new toxin, CRiSP-elapitoxin-Hs2, was identified in peak 15b and made up 0.2% of the venom (Table 1 and Table 2; Figure 8).

**Figure 9:**
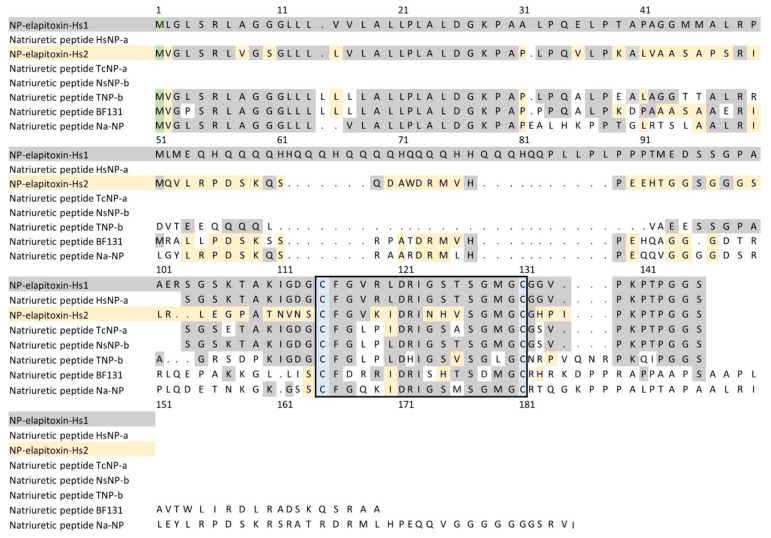
Deduced amino acid sequence for two new ANP/BNP-like natriuretic peptides (NP-elapitoxin-Hs1; row 1; NP-elapitoxin-Hs2; row 3) in the venom of *Hoplocephalus stephensii* compared to a previously identified fragments and sequences of NPs in *H. stephensii* venom (Q3SAE6; row 2), *Tropidechis carinatus* (Q3SAE9; row 4), *Notechis scutatus* (Q3SAE7; row 5), *Oxyuranus scutellatus* (P83228; row 6), *Bungarus flaviceps flaviceps* (D5J9S0; row 7) and *Naja atra* (D9IX97; row 8). Numbering commences at the methionine residue shaded green. Identical residues to NP-elapitoxin-Hs1 are shaded grey and identical residues to NP-elapitoxin-Hs2 are shaded yellow. The 17 amino acid loop of natriuretic peptide is boxed with cysteine residues is highlighted in blue.

There were two protein sequences deduced from the whole-venom MS/MS (unable to be extracted from the individual peaks) for natriuretic peptides. A BLAST search matched the first to a previously identified fragment of a NP from *H. stephensii* venom, and the full sequence we deduced from the transcriptome with propeptide was named NP-elapitoxin-Hs1, which is an atrial/brain-like NP (ANP/BNP-like NP). The second sequence was a new NP named NP-elapitoxin-Hs2 (Figure 9). Because these were only identified in the whole-venom MS/MS, the individual abundances for the two are unknown, but together they make up approximately 0.8% of the venom (Table 1; Figure 4).

Only one partial amino acid sequence was deduced from the MS/MS protein fragments matched to the single transcript found in peak 15a (Table 1). A BLAST search on UniProt closely matched this partial sequence to a complete sequence of the previously identified CTL, C-type lectin galactose-binding isoform. This only confirmed that there was one CTL making up 1.1% of the venom, but insufficient transcript to determine its full sequence.

**Figure 10:**
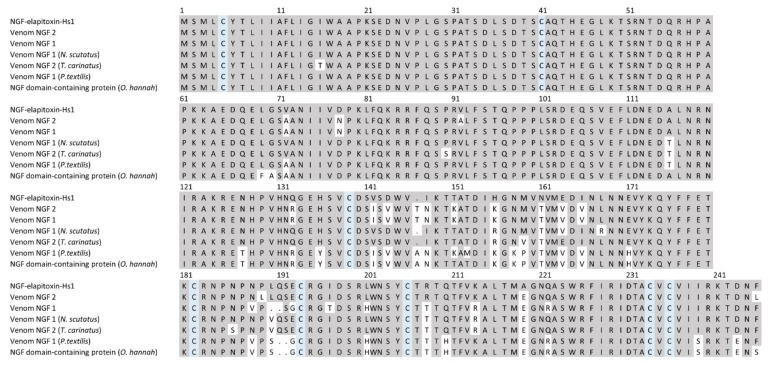
Deduced amino acid sequence for a new NGF, NGF-elapitoxin-Hs1 (row 1), compared to two different NGFs previously identified in *Hoplocephalus stephensii* venom (Q3HXY0; row 2, Q3HXX9; row 3), and NGFs in the venoms of *Notechis scutatus* (Q3HXY7; row 4), *Tropidechis carinatus* (Q3HXX7; row 5), *Pseudonaja textilis* (Q3HXY9; row 6) and *Ophiophagus hannah* (V8NP13; row 7). Conserved cysteine residues are shaded in blue and shaded areas indicate the same residue compared to NGF-elapitoxin-Hs1.

There were three complete and one partial amino acid sequences deduced for a single NGF from MS/MS protein fragments matched to the transcriptome for peaks 12 and 13 (Table 1). A BLAST search on UniProt identified two similar previously identified NGFs found in *H. stephensii* venom, NGF1 and venom NGF2. The NGF we identified was different and named NGF-elapitoxin-Hs1 (Table 1 and Table 2; Figure 10). Using ALIGN in UniProt we demonstrated that NGF-elapitoxin-Hs1 had a sequence much more similar to those found in the closely related species, *N. scutatus* and *T. carinatus*, compared to NGF1 and NGF2 which aligned with *P. textilis* in a different clade and *O. hannah* in a different subfamily (Supplementary Figure S3).

The identified/deduced toxins along with their abundance and peak/band location are summarized in Table 2.

## 3. Discussion

Globally, elapid venoms have been shown to mostly be composed of low-molecular-weight toxins belonging to the protein families 3FTx and/or PLA_2_. Combined, these two dominant toxin families typically make up almost 80% of elapid venoms [19,20]. In contrast, we show that almost half of the venom of *H. stephensii* is made up of high-molecular-weight proteins—SVMP (36.7%) and LAAO (10.8%). The second most abundant protein family in the venom was PLA_2_ (24%), consistent with other elapid venoms, but 3FTx made up only 10% of the venom. Overall, 92% of the protein component of the venom by weight was characterized to the family level, including the four dominant protein families (PLA_2_, 3FTx, SVMP, and SVSP), four secondary families (LAAO, CRiSP, CTL and NP) and one minor family—NGF. Apart from LAAO, the secondary protein families occurred in smaller amounts (<2%). We identified/deduced 18 toxins in 76% of the venom, 14 of which had not previously been characterized or sequenced. Additionally, eight other minor and rare protein families were detected in our whole-venom analysis, which would increase the number of toxins in *H. stephensii* venom to at least 26.

*H. stephensii* venom contains the highest percentage of SVMP and LAAO protein families recorded for an elapid venom to date [19]. Previously, the highest percentage of SVMP and LAAO was 18.7% in *Calliophis bivirgata* venom [23] and 7% in *Bungarus fasciatus* venom, respectively [24]. We confirmed that the high-molecular-weight protein families SVMP and LAAO made up almost half of the venom by two different fractionation methods: chromatography (46%) (Figure 3) and gel electrophoresis (45%) (Supplementary File S2, Figure S4 and Table S2). Compared to the venom proteomes of the four other species of Australian elapids whose venom composition have so far been characterized, the venom of *H. stephensii* is unusual, because the other species with characterized venomes were PLA_2_ dominant (74.5% to 97.6%), and none contained SVMP in amounts greater than 5.2%, or LAAO in amounts greater than 1.6%, [21,25,26,27].

We identified the previously characterized procoagulant toxin Hopsarin D in the venom, except the deduced sequence from the transcriptome differed at residue 3 (signal peptide: histidine instead of a proline) and at residue 70 (light chain: alanine instead of a valine). We also showed that Hopsarin makes up 6% of *H. stephensii* venom, which is more than the finding of a previous study that it only made up <1% of the venom [28]. Hopsarin is the most medically important toxin in *H. stephensii* venom, responsible for venom-induced consumption coagulopathy, the main effect in human envenoming [17]. It is a factor Xa prothrombin activator with a light and heavy chain (Supplementary File S2, Figure S2), and makes up the catalytic subunit of the prothrombinase complex.

Neurotoxicity is a major feature of elapid envenoming in humans worldwide, consistent with the predominance of PLA_2_ and 3FTx in elapid venoms. Neurotoxicity in elapid envenoming can be caused by 3FTx alpha-neurotoxins (e.g., *Naja* spp.), or PLA_2_ presynaptic neurotoxins (e.g., *Bungarus* spp. and several species of Australian elapids). *H. stephensii* does not cause neurotoxicity, which is most likely due to the smaller amounts of 3FTx (Figure 4), and the fact that the major 3FTx, alpha-elapitoxin-Hs1, is a short-chain neurotoxin (Figure 7), which are only associated with human neurotoxicity from some *Naja* spp. and *Ophiophagus hannah* [29,30]. We did identify a long-chain neurotoxin, alpha-elapitoxin-Hs2, but it only made up 1.4% of the venom (Table 2; Figure 7).

We identified and provide the sequence for five different acidic PLA_2_ toxins in *H. stephensii* venom (Figure 6), PLA_2_a-elapitoxin-Hs1 to PLA_2_a-elapitoxin-Hs5, although functional studies are required to determine the effect of these PLA_2_s. Interestingly, PLA_2_a-elapitoxin-Hs4 and PLA_2_a-elapitoxin-Hs5 both have a glutamate (Glu-49) rather than the more common aspartate (Asp-49) at the active site. Whether this affects the catalytic activity of these two Glu-49 PLA_2_s requires further study, but a previous study found that *H. stephensii* only had low PLA_2_ activity [31]. Glutamate has a similar acidic side-chain to aspartate, which may mean these two do retain catalytic activity. This is in contrast to the Lys-49 variant PLA_2_, which does not retain catalytic activity [32].

The high expression level of LAAO in *H. stephensii* is unusual, but high LAAO activity has been recorded for several other genera of Australian elapids including *Demansia, Pseudechis*, and some *Acanthophis* spp [31]. The effect of LAAO in human envenoming remains unclear, and the clinical effects of *H. stephensii* envenoming are mainly explained by Hopsarin.

Our analysis showed low toxin diversity in the venom of *H. stephensii*, with most protein families only containing one or two toxins (Table 2; Figure 4), the exceptions were SVMP, with at least three unique toxins, and PLA_2_, with at least five (Table 2; Figure 5 and Figure 6). Too little is currently known about Australian elapid venoms to determine if this is normal for this elapid radiation. Indeed, toxin diversity is poorly reported for elapids worldwide. In a recent review toxin diversity was only reported in 23 species compared to abundance being recorded in 34 species [20].

Although *H. stephensii* has both a specialized ecology and an unusual venom, this does not necessarily mean that there is a causal relationship or even an association between the two. The divergent venom composition of *H. stephensii* may have been a relatively recent evolutionary development that post-dates the morphological specializations that have evolved in this genus. As there is nothing unique about the diet of *H. stephensii*, and no other elapid has been recorded with such high expression levels of SVMP/LAAO, it raises the possibility that the upregulation of expression of these toxins in the venom of *H. stephensii* may not necessarily have been an adaptation for increased venom lethality. Snakes may only require a small proportion of their venom to be highly potent toxins, and the remainder may contribute little to overall lethality or provide any specific function.

The venom proteome of *H. stephensii* is more difficult to explain in terms of the clinical effects in human envenoming. *H. stephensii* envenoming results in VICC, with no reports of neurotoxicity or myotoxicity [17]. This is consistent with the presence of Hopsarin, a prothrombin activator, and the short-chain 3FTx. Most unusual is the high proportion of SVMP, without any clinical reports of hemorrhagic-like effects, such as spontaneous hemorrhage. This is in stark contrast to true vipers, such as *Echis* spp., in which envenoming causes significant hemorrhagic effects, consistent with the large proportion of SVMPs in their venoms [19,20].

This study used transcriptomic-guided proteomics to give greater resolution of the venom proteome, allowing for the sequences of unique toxins to be assembled and the presence of previously identified toxins in the venom of *H. stephensii* to be confirmed. The benefits of using species-specific transcriptomes are clearly illustrated in this study by our finding of 14 new toxins in the venom of *H. stephensii*, which would not have been possible by solely relying on matching MS derived peptide sequences to a public database. The use of public databases would also have resulted in an underestimation of the true toxin diversity. However, public databases were essential for the initial identification of toxins to the protein family level.

Limitations to our study include only characterizing 92% of the venom. There is also a need to clarify the precise number of SVMPs and PLA_2_s present. Additionally, elucidating the evolutionary cause or lack of restraint on the expression levels of SVMP and LAAO could shed light on how snake venom proteomes become altered. A further line of research could be investigating if any of the PLA_2_s occur as oligomers, as has been recorded in some other Australian elapids such as *Oxyuranus scutellatus* [33], *Acanthophis antarcticus* [34], and *Pseudechis colletti* [27]. The medical importance of many of the toxins in *H. stephensii* venom, like most snake venoms, remains unclear. Most of the clinical effects seen in human envenoming can be explained by the effects of Hopsarin, which only makes up 6% of the venom. The remaining toxins may play some role in prey capture, but further functional studies in prey species need to be undertaken.

## 4. Methods

### 4.1. RNA Extraction

An adult female *Hoplocephalus stephensii* specimen was collected from Cooranbong (Watagan Mountains) New South Wales (N.S.W), Australia. To create the transcriptome assembly for *H. stephensii*, the snake was anaesthetized four days after milking, then euthanized and the venom glands removed and stored in RNAlater (ThermoFisher Scientific, Macquarie Park, N.S.W. Australia). The snake was anesthetized with isoflurane and euthanized with sodium pentobarbitone. A maximum of 30 mg of tissue was excised from the sample and then disrupted and homogenized using a TissueLyser II (Qiagen, Hilden, Germany). RNA was extracted using a RNeasy Plus Universal Midi Kit (Qiagen) following the manufacturer’s protocol. RNA quality was ascertained on a Bioanalyser (Agilent Technologies, Santa Clara, CA, USA) a RNA Integrity number (RIN) (above 8.0) indicating the sample was of appropriate quality for downstream transcriptomic and sequencing analysis. The venom of the same snake was also used for whole-venom analysis and in the pooled venom for the fractionation and bottom-up proteomics.

### 4.2. Quality Assessment of Raw Reads

FASTQC (Babraham Bioinformatics, Cambridge, UK) [35] was used to assess the quality of the raw reads using a k-mer size of 7. Additionally, one thousand raw reads were randomly selected and aligned to the non-redundant nucleotide database at National Centre for Biotechnology Information (NCBI) (Bethesda, MD, USA) with Blast+ v2.6.0 [36]

### 4.3. Transcriptome Assembly

Transcriptome assembly was performed using pooled reads and following the protocol described by Cerveau and Jackson [37]. Briefly, three independent assemblies were performed with Trinity [38] (Kmer = 55), Oases [39] (Kmer = 43, 53, 63, 73) and Shannon [40] (Kmer = 65). Oases assemblies from K = 53, 63 and 73 were merged into a single oases transcriptome assembly following oases recommendations. Results from each of the three assemblies were clustered with CD-HIT-EST [41] with the following parameters: G 0, c 1.00, aS 1.00, and aL 0.005. Representative transcripts from each assembly were then pooled and Transdecoder [42] was used to extract open reading frames (ORF) longer than 100 aa. The coding sequences were further clustered using CD-HIT-EST with the following parameters: c 0.98, G 0, M 16,000, T 16, aS 1.0, and aL 0.05. Representative coding sequences were used as annotation for downstream analysis and the contigs where they originated were used as the final transcriptome assembly.

### 4.4. Assembly Validation

BUSCO v3 [43] was used to identify universal single copy orthologs and to estimate the completeness of the assembly using the Metazoa database v 9. Additionally, reads were mapped back to the assembly with Bowtie 2 v 2.3.2 and mapping efficiency was recorded.

### 4.5. Differential Gene Expression

The alignments obtained from the previous step were sorted by coordinates using Samtools 1.6 [44]. The raw count of reads per gene feature were calculated using the featureCounts v1.4.6-p5 utility of the subread package (http://subread.sourceforge.net/ (accessed on 15 February 2018)). EdgeR v. 3.16.5 [45] package was used to perform differential expression analysis (https://bioconductor.org/packages/release/bioc/html/edgeR.html (accessed on 15 February 2018)). The default Trimmed Mean of M-values (TMM), normalization method of edgeR was used to normalize the counts. Generalised Linear Model (GLM), model was used to perform differential expression comparison between the groups. Finally, differentially expressed genes were classified as either active or inactive in the venom gland tissue based on its count per million reads (cpm). If a gene had 0 cpm in both repeats of the same tissue, it was classified as inactive (not expressed) in that tissue.

### 4.6. Functional Annotation

Peptide sequences were obtained from the transcriptome with the software TransDecoder [42]. Peptide sequences longer than 100 residues were kept for further annotation. InterProScan v5.28-67.0 [46] was used to perform functional annotation of the peptides using default parameters. 

### 4.7. Toxin Identification and Classification

After functional annotation, additional proteins were assigned to known snake venom protein families from a comprehensive published review [19] based on orthologous group information produced by OrthoMCL [47]. Briefly, if an annotated protein was assigned to an orthologous group by OrthoMCL, then all proteins in the same group were also classified to the same function. 

### 4.8. Comparison of Expression Levels and Gene Counts

Only genes with a normalized read count greater or equal to 1 (cpm ≥ 1) in both replicates of the venom gland libraries were taken into account for gene count and comparisons. Pie charts were produced to compare total gene count in the venom gland transcriptome and normalized read counts in the venom gland.

### 4.9. Orthologous Identification

Transcript and protein sequence data from the king cobra genome (*O.s hannah*) were downloaded from the NCBI genome database (https://www.ncbi.nlm.nih.gov/genome/?term=cobra (accessed on 15 February 2018). The transcriptome assembly produced was converted into proteins using TransDecoder v0.5.1 [42] and the longest ORFs were kept as representative proteins for each transcript. OrthoMCL v 2.0.9 [47] was used to identify orthologous proteins using default parameters and following the developer’s recommendation. Blast+ v2.7.1 [36] was used to determine similarity for OrthoMCL analysis.

### 4.10. Mapping Genes to the Cobra Genome Assembly

Based on the OrthoMCL analysis, genes that were unequivocally mapped to a single locus in the Cobra genome assembly were selected and the coordinates extracted from the Cobra genome annotation file. Correlation was made for the OrthoMCL group with cobra gene, their corresponding orthologues in the assembled transcriptomes and their genome coordinates.

### 4.11. Venom

*H. stephensii* venom was collected from two adult female snakes from Bungwahl and Cooranbong N.S.W, respectively These localities are 115 km apart on the central coast of N.S.W at the southern end of the species distribution. Both snakes were maintained in captivity at Venom Supplies Tanunda South Australia S.A.

### 4.12. Reverse-Phase High-Performance Liquid Chromatography

A Shimadzu LC–20AD (Shimadzu, Scientific Instruments, Sydney, Australia), pump was used and peak elution was monitored with a Shimadzu SPD-20A detector set at an absorbance of 214 nm. Lyophilized whole venom was reconstituted in Millipore water (Merk Millipore, Burlington, MA, USA) at a concentration of 10 mg/mL for protein recovery for 1D SDS-PAGE. Injection volume was 200 µL. The method used was as follows: Mobile phase A was water with 0.1% trifluoroacetic acid (TFA), mobile phase B was 100% acetonitrile with 0.1% TFA. Flow rate was 1 mL/min. The gradient used was 0% to 5% over 10 min, 5% to 15% over20 min, then 15% to 45% over 120 min, then 45% to 70% over 20 min, and finally 70% to 100% over 15 min. Software processing and peak integration was done using LabSolutions (2010–2017 Shimadzu Corporation, Sydney, Australia). After collection, the venom fractions were frozen at −80 °C and then lyophilized on a 4.5 L −105 °C freeze dryer (Labconco, Kansas City, Missouri).

### 4.13. One Dimensional (1D) SDS-PAGE

Electrophoresis was performed using 4–20% Mini Protean TGX gels (Bio Rad Laboratories, Gladesville, N.S.W. Australia). Each well was loaded with 16 µL of a 3:1 (*v*/*v*) ratio of venom/sample buffer. The whole-venom concentration was 1 mg/mL giving 12 µg of venom per well. Lyophilized chromatographic fractions were reconstituted with 20 µL of Millipore water for small peaks which was increased up to approximately 50 µL for very large peaks to avoid overloading the gel. Reducing conditions were 95°C for 4 min. Gels were run at 170 V until the dye front was within 10 mm of the base of the gel. Gels were then fixed for 1 h in a fixing solution of (5:4:1) methanol/water/acetic acid, then stained with Coomassie Blue dye for 1 h. The gels were then destained in Millipore water for several days. The gels were imaged with a Bio Rad Gel Doc Go Imaging System (Bio-Rad Laboratories) which was also used to perform densitometry.

### 4.14. Bottom-up Proteomics

#### 4.14.1. In-Gel Trypsin Digestion

In-gel trypsin-digested venom fractions were prepared for bottom-up proteomic analysis. A single gel plug from each of the stained gel bands was excised and placed in separate wells in a 96-well microplate. Gel plugs were destained with 50% methanol solution for 1 h twice and then once with a 70% methanol solution for 1 h. A volume of 100 µL of 10 mM dithiothreitol (DTT) in 25 mM ammonium bicarbonate (AB) was added to each well for 30 min. Wells were then rinsed with AB and 100 µL of 50 mM iodoacetamide (IAA) in AB was added to each well for 30 min. Wells were then rinsed in AB and 100% acetonitrile (ACN), was added for 15 min. A volume of 25 µL of trypsin solution (trypsin 1 µg per 250 µL of AB stock), was added to each well. The plate was incubated at 37 °C for 2 h and then left at room temperature overnight to digest. Peptides were extracted with triplicate washes of 100 µL of 50% ACN with 0.1% TFA. Washes were pooled into 1.5 mL microcentrifuge tubes and freeze-dried for later resuspension in 0.1% formic acid.

#### 4.14.2. In-Solution Trypsin Digestion

For liquid fractions, filter-aided trypsin digestion was performed in Amicon Ultra-0.5 mL centrifugal filter units (Merk Millipore, Burlington, MA, USA) with a 3 kDa-molecular-weight cut-off. Lyophilized venom was used in amounts ranging from 0.1 to 0.38 mg. Venom proteins were first reconstituted in 200 μL of 7 M urea/100 mM ammonium bicarbonate (AB) and centrifuged at 14,000× *g* for 45 min. Samples were then incubated with 100 μL of 50 mM Dithiothreitol (DTT) in 7 M urea/100 mM NH4HCO3 at room temperature for 1 h. DTT was removed by centrifugation for 1 h at 14,000× *g*. Samples were incubated with 100 μL of 55 mM Iodoacetamide (IAA) in 7 M urea/100 mM AB for 20 min in darkness prior to centrifugation at 14,000× *g* for 45 min. Promega MS grade trypsin (ThermoFisher Scientific, Macquarie Park, N.S.W. Australia), resuspended at 100 ng/μL in 10 mMAB, was added to the samples at a mass ratio of 1:50 (trypsin: protein). The samples were incubated at 37 °C overnight. Digested peptides were eluted through the spin-filter and dried using vacuum centrifugation prior to reconstitution in 100 μL of 2% ACN and 0.1% formic acid (FA). The samples were then purified with a C18 Biospin column (ThermoFisher Scientific). Concentrations were verified on a NanoDrop 2000/2000c UV–Vis spectrophotometer (ThermoFisher Scientific), at a wavelength of 205 nm using an extinction coefficient, ε205, of 31 mL mg^−1^ cm^−1^. Samples were stored at −20 °C until required for LC–MS/MS analysis.

#### 4.14.3. LC–MS/MS Analysis of Venom Fractions

Nano-LC–ESI–MS/MS was performed on the venom fractions using an Ultimate 3000 RSLC system (Thermo Fisher Scientific) coupled to a LTQ Orbitrap XL ETD mass spectrometer (Thermo-Fisher Scientific). Approximately 1.5 µg of each peptide sample was first pre-concentrated on a C18 trapping column (Acclaim PepMap 100 C18 75 µm × 20 mm, Thermo-Fisher Scientific) at a flow rate of 5 µL/min using 2% (*v*/*v*) acetonitrile 0.1% (*v*/*v*) trifluoroacetic acid for 10 min. Peptides were then separated using a 75 µm ID C18 column (Acclaim PepMap100 C18 75 µm × 50 cm, Thermo-Fisher Scientific) at a flow rate of 0.3 µL/min, using a linear gradient of 5% to 45% solvent B over 38 min. This was followed by a 2 min wash with 90% Solvent B, and then a 15 min equilibration process with 5% Solvent B. (Solvent A: 2% (*v*/*v*) acetonitrile, 0.1% (*v*/*v*) formic acid. Solvent B: 80% (*v*/*v*) acetonitrile 0.1% (*v*/*v*) formic acid). LC–MS/MS acquisition were performed in the data-dependent acquisition mode. The conditions used were as follows: *m*/*z* range, 300–2000; polarity, positive. MS1 scans were acquired at a resolution of 60,000 in FT mode. The 5 most intense precursor ions were selected for isolation and subjected to CID fragmentation using a dynamic exclusion of 5 s. Dynamic exclusion criteria included a minimum relative signal intensity of 1000, and ≥2+ charge state. An isolation width of 3.0 *m*/*z* was used with a normalized collision energy of 35.

#### 4.14.4. Identification of Toxins Using PEAKS Software and Uniprot

Settings for PEAKS (Bioinformatics Solutions Inc, Waterloo, Canada), were; charge between 1 and 8, PTM modifications—cysteine carbamidomethylation and methionine oxidation. Maximum variable PTM was 3. Precursor mass tolerance was set at 50.0 ppm using monoisotopic mass and fragment ion mass tolerance was set at 0.6 Da. Filter search parameters were set at a minimum of 2 unique peptides, however one protein family (CTL—1.1%), could only be matched by lowering the search parameter to 1 unique peptide. False Discovery Rate (FDR) was set for 1, and Proteins −10lgP was set to the same value as FDR.

The peptide sequences derived from bottom-up proteomics were matched to complete peptide sequences derived from the assembled species-specific transcriptome. Using a BLAST search on UniProt the complete peptide sequences were then matched to either a protein family based on a high sequence similarity to a known toxin, or in some cases to a specific unique toxin if known toxin sequences were taken from our assembled transcriptome and aligned manually.

## Figures and Tables

**Table 1:** Protein families identified in each chromatogram peak based on sequence similarity to matches on UniProt (see Section 2.2.3). Relative abundances were calculated by either integration of chromatogram peaks, or a combination of peak integration, densitometry and spectral intensity (MS1). Band designations a, b, and c are the locations on the gel lanes for peaks containing more than one band. Peak/band 5b * was based on RP-HPLC and SDS-PAGE fractionation only, as the peak overlapped with peak 4 and both peaks had almost identical gel electrophoretic profiles and band b location.

Peak No #	Band Designation	Protein Family	Peak % of Whole Venom (WV) (Integration)	Band % of Peak (Densitometry)	Toxin % of Band (MS1)	Protein Family % of WV
1		3FTx	4.2	100		4.2
2		3FTx	4.6	100		4.6
3		3FTx	1.4	100		1.4
4			1.7			
	4b	PLA2		55		0.93
5			0.9			
	5b *	PLA2		39		0.35
6			0.7			
	6b	PLA2		66.5		0.46
7			4.3			
	7a			16.5		0.7
	7b	PLA2		83.5		3.59
8			6.0			6.0
	8a	PLA2		15.33		
	8b	PLA2		84.67		
9			3.2			3.2
	9a	PLA2		46		
	9b	PLA2		54		
10		PLA2	0.5	100		0.5
11		PLA2	8.0	100		8.0
12		NGF	0.8	74.5	69.05	0.41
		PLA2			30.94	0.18
13		NGF	0.5	82.9	94.74	0.39
14			7.6			
	14a	SVSP		27.7	84	1.76
		PLA2			5.86	0.12
		CRiSP			8.68	0.18
	14b	CRiSP		18		1.34
	14c	SVSP		54.3		4.13
15			1.7			
	15a	CTL		74		1.08
	15b	CRiSP		26	50.6	0.22
16		SVMP	1.2	100		1.2
17		SVMP	0.7	100		0.7
18		SVMP	10.1	100		10.1
19		LAAO	18.9	100	44.14	8.34
		SVMP			55.86	10.53
20			15.5			
	20a	LAAO		19.72	13.8	0.42
		SVMP			86.2	2.63
	20b	SVMP		42		6.51
	20c	LAAO		38.28	33.67	1.99
		SVMP			66.33	3.93
21		SVMP	1.1	100		1.1

**Table 2:** List of toxins recorded from the venom of *Hoplocephalus stephensii*, the peak and fraction they occurred in (Peaks 1 to 21 and some peaks further divided into 3 bands—a, b and c), their percentage of the whole venom, and their peak occurrence.

Protein Family/Toxin	% of Whole-Venom Composition	Peaks/Bands
**3FTx**		
alpha-elapitoxin-Hs1 (short-chain neurotoxin)	8.8	1,2
alpha-elapitoxin-Hs2 (long-chain neurotoxin)	1.4	3
**PLA_2_**		
PLA_2_a-elapitoxin-Hs1	8.5	10, 11, 12
PLA_2_a-elapitoxin-Hs2	0.5	9a
PLA_2_a-elapitoxin-Hs3	5.9	8b, 9a
PLA_2_a-elapitoxin-Hs4	1.7	9b
PLA_2_a-elapitoxin-Hs5	3.2	7
**SVMP**		
SVMP-III-elapitoxin-Hs1	10.1	18
SVMP-III-elapitoxin-Hs2	10.53	19
SVMP-III-elapitoxin-Hs3	6.51	20b
**SVSP**		
Hopsarin D	5.9	14a, 14c
**LAAO**		
LAAO-elapitoxin-Hs-1	10.33	19 20c
LAAO-elapitoxin-Hs-1 proteoform	0.42	20a
**CRiSP**		
CRiSP-elapitoxin-Hs-1	1.52	14a,14b
CRiSP-elapitoxin-Hs-2	0.22	15b
**CTL**		
unsequenced	1.08	15a
NGF		
NGF-elapitoxin-Hs1	0.8	12,13
NP	0.79	4, 5, 6
NP-elapitoxin-Hs1		
NP-elapitoxin-Hs2		

## Data Availability

Not applicable.

## Supplementary Materials

The following supporting information can be downloaded at: https://www.mdpi.com/article/10.3390/toxins14050314/s1, Figure S1: Deduced amino acid sequences for a new LAAO toxin (LAAO-elapitoxin-Hs1; row 1) and a proteoform of this differing by four residues (yellow shaded; row 2) found in *H. stephensii* venom compared to the previously identified L-amino-acid-oxidases in *N. scutatus* venom (Q4JHE2; row 3), *P. australis* venom (Q4JHE1; row 4), *B. fasciatus* (A8QL52; row 5) and *Calloselasma rhodostoma* (P81382; row 6). The signal sequence is shaded orange, conserved cysteine residues are shaded in blue and shaded grey areas indicate the same residue compared to LAAO-elapitoxin-Hs1. Figure S2: Seven deduced protein sequences from the transcriptome after matching with fragments from MS/MS, which were then matched using a BLAST search in UniProt to the previously identified hopsarin (P83370). The domains are shaded as follows: signal peptide (red), propeptide (yellow), light chain (blue), activation peptide (grey) and heavy chain (green), with non-shaded areas indicating different residues, importantly residues 3 and 70 that differ to hopsarin. Regions of the deduced sequence where MS/MS protein fragments matched are in bold blue (light chain) and bold green (heavy chain). Figure S3: Tree diagram of the seven sequences for NGFs from Figure 10 using ALIGN in UniProt. This indicates that the new NGF-elapitoxin-Hs1 (transcript id: Shannon_61405) we identified is most similar to two previously identified NGFs in the closely related species, *N. scutatus* (NGFV1_NOTSC) and *T. carinatus* (NGFV2_TROCA). The previously identified NGFs in *H. stephensii* venom (NGFV1_HOPST and NGFV2_HOPST), have sequences closer to *P. textilis* (NGFV1_PSETE) and *O. hannah* (V8NP13_OPHHA), species in a different clade and a different subfamily, respectively. Figure S4: SDS-PAGE analysis of four elapid snake venoms shows the unusually high proportion of high molecular weight toxins (60 to 90kDa), in the venom of *Hoplocephalus stephensii* (pooled from two individuals), compared to elapid species with “typical” elapid 3FTx or PLA_2_ (5 to 15kDa) dominant venoms; Monocled Cobra *Naja kaouthia*, Southern death adder *Acanthophis antarcticus*, and Papuan black snake *Pseudechis papuanus*. Table S1: Protein families detected by in-solution digestion of the whole venom. Asterisks indicate protein families not detected by venom fractionation and in-gel trypsin digestion. Table S2: Densitometry of the whole venom of *Hoplocephalus stephensii* (taken from Figure S3)*,* showing the high molecular weight toxins SVMP and LAAO make up 45% of the whole venom when fractionated electrophoretically.
