# Supplementary material for: The Unusual Metalloprotease-Rich Venom Proteome of the Australian Elapid Snake Hoplocephalus stephensii
Source: Toxins (Basel). 2022 Apr 28;14(5):314. doi: 10.3390/toxins14050314 (PMC9147224; doi:10.3390/toxins14050314)
Supplement: Supplementary file 1 [file toxins-14-00314-s001.zip › Supplementary File S2.pdf]

toxins

MDPI

Supplementary Materials

The Unusual Metalloprotease-Rich Venom Proteome of the Australian Elapid Snake *Hoplocephalus stephensii*

Theo Tasoulis [1,*], C. Ruth Wang [2], Joanna Sumner [3], Nathan Dunstan [4], Tara L. Pukala [2] and Geoffrey K. Isbister [1]

[1] Clinical Toxicology Research Group Newcastle, University of Newcastle, Newcastle, NSW 2308, Australia; geoff.isbister@gmail.com
[2] Department of Chemistry, Faculty of Sciences, University of Adelaide, Adelaide, SA 5005, Australia; chia-de.wang@adelaide.edu.au (C.R.W.); tara.pukala@adelaide.edu.au (T.L.P.)
[3] Genetic Resources, Museums Victoria, Carlton Gardens, Melbourne, VIC 5053, Australia; jsumner@museum.vic.gov.au
[4] Venom Supplies, Tanunda, SA 5352, Australia; venoms@venomsupplies.com
* Correspondence: theo.tasoulis@newcastle.edu.au

Table S1. Protein families detected by in-solution digestion of the whole venom. Asterisks indicate protein families not detected by venom fractionation and in-gel trypsin digestion.

**3FTx**
PLA2
SVSP
SVMP
CRiSP
CTL
LAAO
Natriuretic peptide*
NGF
5′Nucleotidase*
Glutaminyl cyclase*
Hyaluronidase*
Phosphatase*
Phospholipase B*
Sulfhydryl oxidase*
Waprin*
Aminopeptidase*

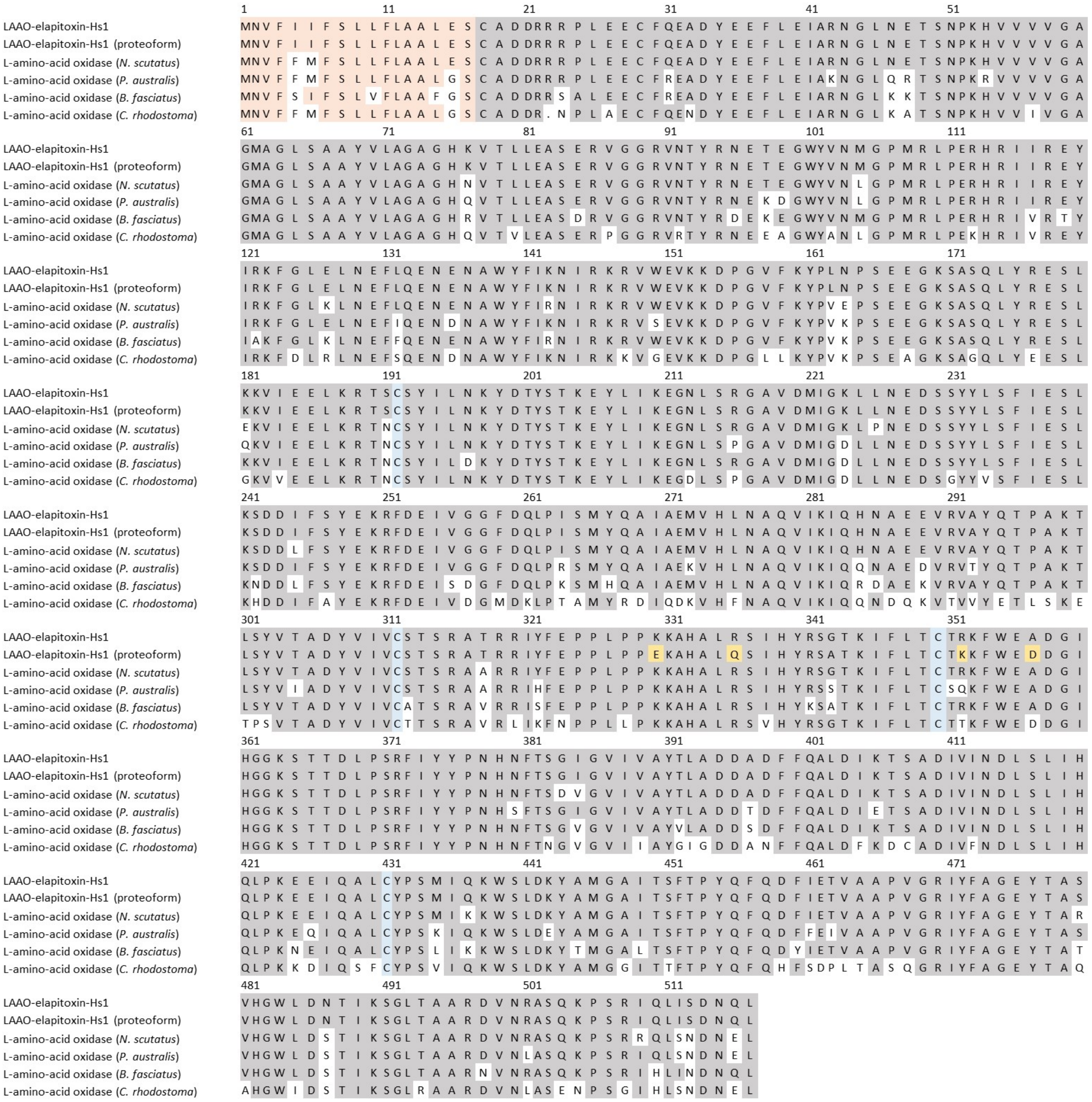

Figure S1. Deduced amino acid sequences for a new LAAO toxin (LAAO-elapitoxin-Hs1; row 1) and a proteoform of this differing by four residues (yellow shaded; row 2) found in *H. stephensii* venom compared to the previously identified L-amino-acid-oxidases in *N. scutatus* venom (Q4JHE2; row 3), *P. australis* venom (Q4JHE1; row 4), *B. fasciatus* (A8QL52; row 5) and *Calloselasma rhodostoma* (P81382; row 6). The signal sequence is shaded orange, conserved cysteine residues are shaded in blue and shaded grey areas indicate the same residue compared to LAAO-elapitoxin-Hs1.

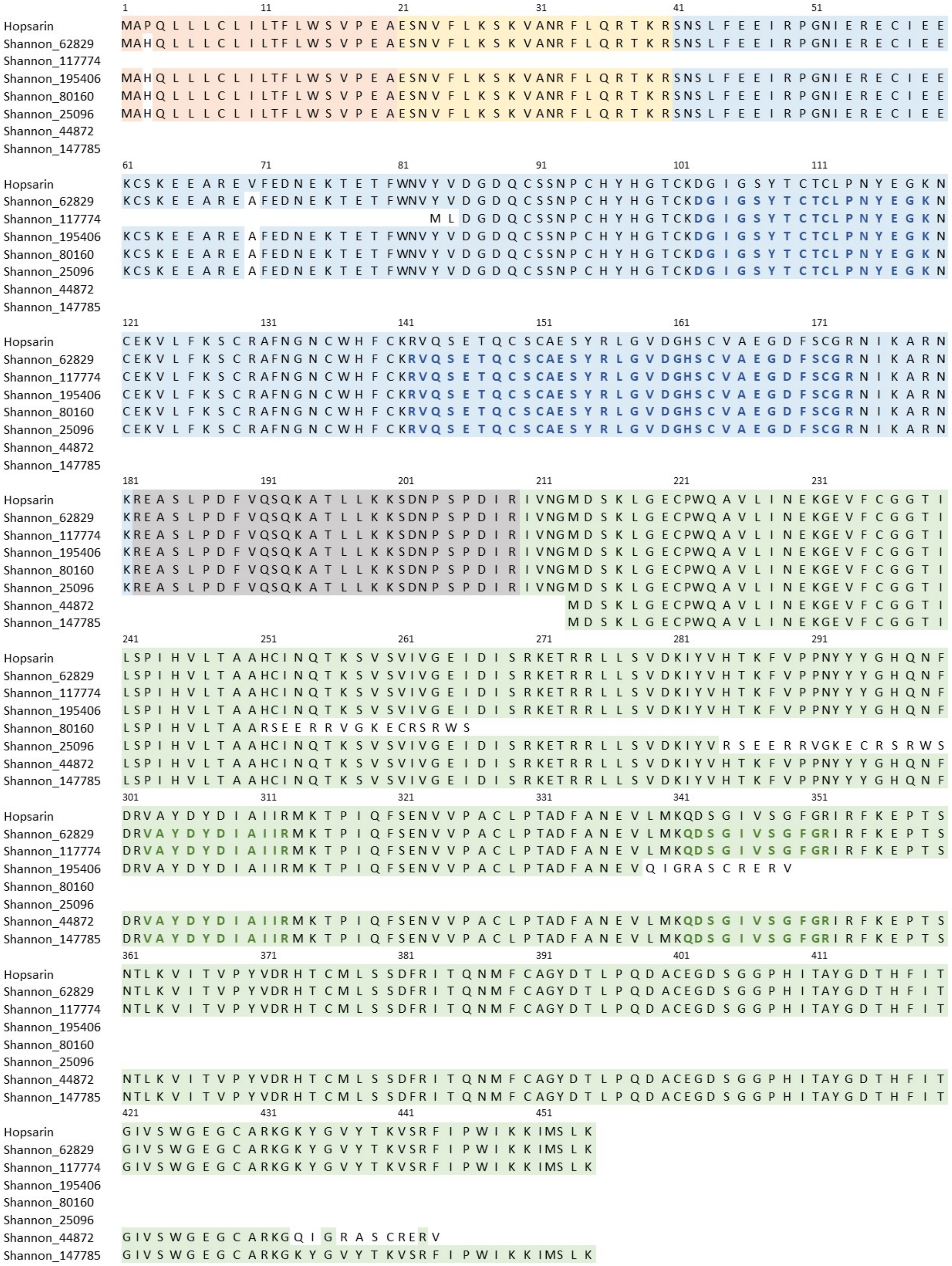

Figure S2. Seven deduced protein sequences from the transcriptome after matching with fragments from MS/MS, which were then matched using a BLAST search in UniProt to the previously identified hopsarin (P83370). The domains are shaded as follows: signal peptide (red), propeptide (yellow), light chain (blue), activation peptide (grey) and heavy chain (green), with non-shaded areas indicating different residues, importantly residues 3 and 70 that differ to hopsarin. Regions of the deduced sequence where MS/MS protein fragments matched are in bold blue (light chain) and bold green (heavy chain).

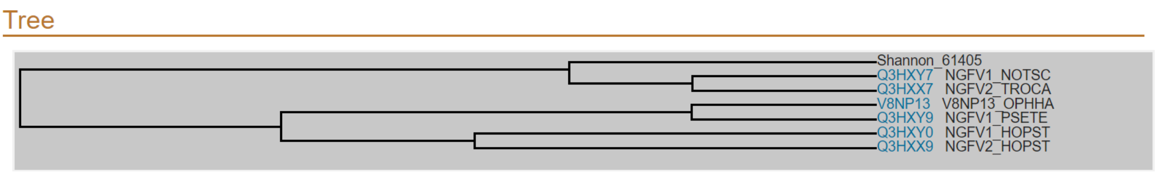

Figure S3. Tree diagram of the seven sequences for NGFs from Figure 10 using ALIGN in UniProt. This indicates that the new NGF-elapitoxin-Hs1 (transcript id: Shannon_61405) we identified is most similar to two previously identified NGFs in the closely related species, *N. scutatus* (NGFV1_NOTSC) and *T. carinatus* (NGFV2_TROCA). The previously identified NGFs in *H. stephensii* venom (NGFV1_HOPST and NGFV2_HOPST), have sequences closer to *P. textilis* (NGFV1_PSETE) and *O. hannah* (V8NP13_OPHHA), species in a different clade and a different subfamily, respectively. .

The crude venom of *H. stephensii* (pooled from two individuals), was first subjected to sodium dodecyl sulfate polyacrylamide gel electrophoresis (SDS-PAGE) analysis (Figure S4). The electrophoretic venom profile was compared with an Asian elapid – the monocled cobra *Naja kaouthia*, and two other species of Australian elapids – Southern death adder *Acanthophis antarcticus* and the Papuan black snake *Pseudechis papuanus* (Figure S4). A remarkable feature of the venom profile of H. *stephensii* was the unusually high abundance of high molecular weight toxins in the mass range of 60 to 90kDa. This was calculated by densitometry to be 45% of the whole venom (Table S2).

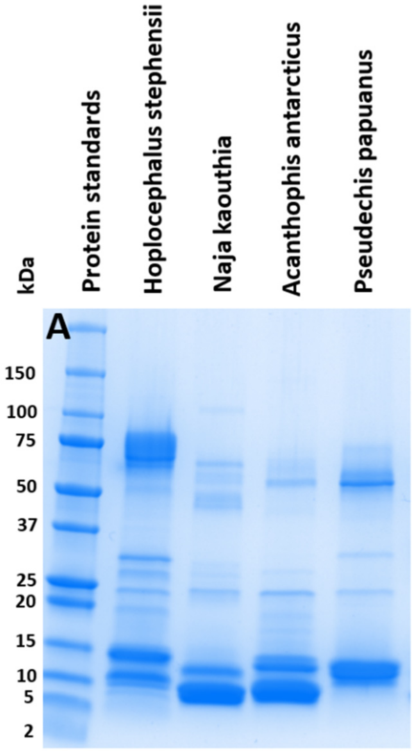

Figure S4. SDS-PAGE analysis of four elapid snake venoms shows the unusually high proportion of high molecular weight toxins (60 to 90kDa), in the venom of *Hoplocephalus stephensii* (pooled from two individuals), compared to elapid species with "typical" elapid 3FTx or PLA_2 (5 to 15kDa) dominant venoms; Monocled Cobra *Naja kaouthia*, Southern death adder *Acanthophis antarcticus*, and Papuan black snake *Pseudechis papuanus*.

Table S2. Densitometry of the whole venom of *Hoplocephalus stephensii* (taken from Figure S3), showing the high molecular weight toxins SVMP and LAAO make up 45% of the whole venom when fractionated electrophoretically.

Densitometry of WV Pooled 2 Individuals				
Lane	Band No.	Band %		
1	1	44.62928	45%	high molecular weight
1	2	3.842845		
1	3	2.24096		
1	4	2.058681		
1	5	2.122584		
1	6	22.29095		
1	7	18.2846		
1	8	4.530104		
